# Preoperative neoadjuvant targeted therapy with apatinib for inoperable differentiated thyroid cancer: A case report

**DOI:** 10.1097/MD.0000000000025191

**Published:** 2021-03-26

**Authors:** Yingchao Zhang, Xianzhao Deng, Zheng Ding, Jie Kang, Bo Wu, Bomin Guo, Youben Fan

**Affiliations:** a Center of Thyroid and Parathyroid, Department of Thyroid-breast-hernia Surgery, Shanghai Jiao Tong University Affiliated Sixth People's Hospital; b The International Peace Maternity and Child Health Hospital, School of Medicine, Shanghai Jiao Tong University, Shanghai, China.

**Keywords:** apatinib, case report, differentiated thyroid cancer, locally advanced thyroid cancer, neoadjuvant therapy

## Abstract

**Rationale:**

Though the majority of differentiated thyroid cancer (DTC) patients have a good prognosis after careful and standardized therapy, approximately 13% to 15% of DTC cases show surprisingly aggressive behavior and invasion of the surrounding structures, and a few progress to unresectable diseases. In this study, we report a case of an inoperable locally advanced DTC patient who underwent a curative operation after treatment of preoperative monotherapy of apatinib in a short time.

**Patient concerns:**

A 64-year-old woman complained of dysphagia due to large cervical mass, which severely invaded the left esophagus at the junction of the neck and thorax.

**Diagnoses:**

The female patient was diagnosed with locally advanced papillary thyroid cancer (PTC) by cytopathology and it was difficult to perform a safe and complete removal.

**Interventions:**

Apatinib (500 mg orally once a day) was initially used to treat this patient as a neoadjuvant therapy.

**Outcomes:**

Six weeks later, the tumor dramatically shrunk from 56 × 37 mm to 29 × 26 mm with well-controlled mild hypertension. After a 10-day interval of apatinib withdrawal, complete tumor excision was accomplished through cervical incision without esophageal fistula. Postoperative thyroid stimulating hormone suppression and radioiodine ^131^I ablation therapy were performed. At the 1-year follow-up evaluation, no tumor recurrence or metastasis was observed.

**Lessons:**

Preoperative short term targeted treatment with apatinib for locally advanced inoperable DTC may become a promising neoadjuvant therapy that, can reduce the tumor size and decrease stage, thus making the complete and safe removal of the lesion feasible.

## 1 Introduction

Thyroid cancer (TC) is the most common endocrine malignancy and caused 41,000 deaths worldwide in 2018.^[1]^ Differentiated thyroid cancer (DTC) refers to neoplasms derived from thyroid follicular epithelial cells, and consists of papillary thyroid cancer (PTC), follicular thyroid cancer (FTC), Hurthle cell thyroid cancer (HCTC), and poorly differentiated thyroid cancer (PDTC).^[2]^ DTC comprises the vast majority (>90%) of all TC,^[3]^ and generally carries good prognoses after standard surgery with thyroid stimulating hormone (TSH) endocrine inhibition and sometimes also with radioactive iodine (RAI) therapy. Nevertheless, approximately 13% to 15% of DTC cases could show surprisingly aggressive behavior and involve the surrounding structures, such as the recurrent laryngeal nerve (RLN), trachea, esophagus, larynx, pharynx, or major cervical vessels, which complicates the surgical treatment and dramatically increases postoperative complications and mortality or even leads to tumor progression to unresectable disease.^[4]^

In fact, few guidelines are dedicated to the optimal approach of these locally advanced TCs.^[5]^ Several kinds of methods can be applied, such as external beam radiation therapy (EBRT), systemic chemotherapy (ChT), molecularly targeted treatment, radioactive ^125^I seed implantation, chemoembolization, thermal (radiofrequency or cryo-) ablation, and ethanol ablation, but complete and adequate surgery for TC represents the main-stay of the multimodal treatment approach and the most significant determinant of outcome.^[3]^ Nevertheless, the surgical management of advanced TC is complex and challenging, and in some cases, it is difficult or impossible to perform an effective surgery. In that specific context, how can we improve the clinical stage and create an opportunity for surgery? Similar to breast-conserving surgery or anus-preserving operation combined with preoperative chemotherapy for the treatment of middle-advanced cancer, neoadjuvant therapy would be worthwhile to try in DTC.

Pathological angiogenesis is an important characteristic and essential for tumor growth, invasion, and metastasis, especially for solid tumors.^[6]^ Vascular endothelial growth factor (VEGF) and its receptor are considered potent factors in angiogenesis and are excessively expressed in solid tumors; thus, they have been confirmed to be promising targets for the treatment of neoplasms. Apatinib is a novel, oral, small-molecule tyrosine kinase inhibitor (TKI) that highly and selectively targets vascular endothelial growth factor receptor 2 (VEGFR-2). Recent clinical trials have revealed its hopeful anticancer effect in various solid tumors,^[7,8]^ and a similar result in TC was found in our team's previous research.^[9]^ Herein, we report a case of a locally advanced inoperable DTC patient who underwent complete safe removal after undergoing preoperative short term apatinib targeted treatment, with little drug-related side effects and almost no postoperative complications.

## 2 Case presentation

The study was approved by the Ethics Committee of Shanghai Jiao Tong University Affiliated Sixth People's Hospital. Written informed consent was obtained from the participant. A 64-year-old woman was admitted to our hospital, who complained of gradually worsening dysphagia for half a year and could currently only intake fluids. This patient was diagnosed with PTC by cytopathology at the local hospital and it was difficult to perform total surgical resection due to the severe tumor invasion of the esophagus in the cervicothoracic junction. She had no history of high blood pressure, diabetes, hyperthyroidism, autoimmune diseases, or family illness. After physical examination, we found a large hard and immobile mass located in the left neck (7 × 6 cm in size). The hormone levels of thyroid function were normal except for a significant increase in thyroglobulin (Tg) (1364.00 ng/mL; reference range, 3.50–77.00 ng/mL).

**Figure 1:**
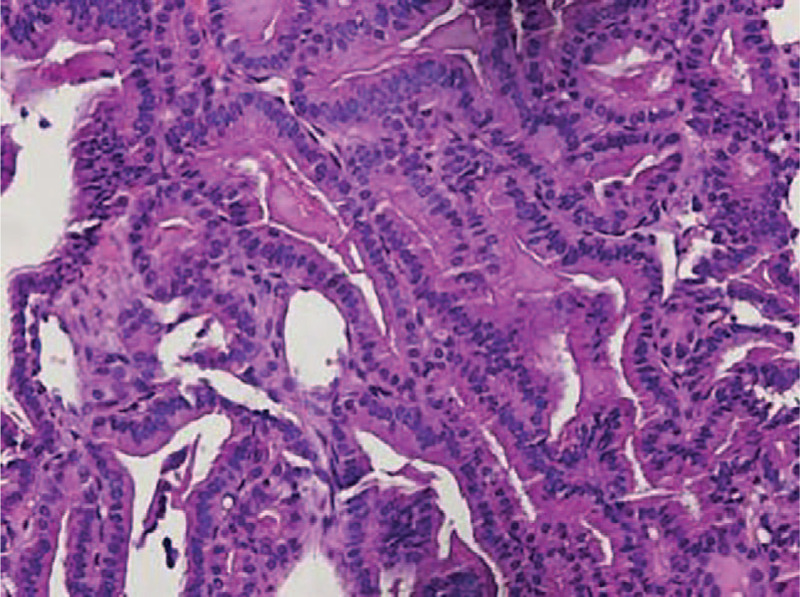
Biopsy highlighting the papillary growth pattern (hematoxylin and eosin staining, original magnification × 100).

**Figure 2:**
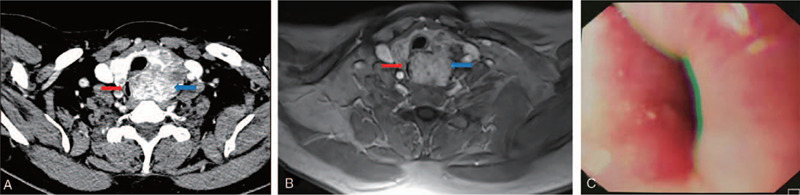
Initial examinations revealed a huge left thyroid mass with esophageal stenosis and invasion (the red arrow indicates the esophagus, and the blue arrow indicates the tumor). (A) CT scan. (B) MRI scan. (C) Esophagogastroduodenoscopy. CT = computed tomography, MRI = magnetic resonance imaging.

A repeat ultrasound inspection revealed a 36 × 39 × 73 mm hypoechoic mass with irregular modality, an ill-defined border, heterogeneous internal echoes, and an abundant color flow signal in the left neck. Moreover, there was no enlarged cervical or supraclavicular lymphadenopathy. Ultrasound-guided core biopsy in the left lobe of the thyroid gland was performed, and the pathology indicated classic PTC (Fig. 1). A contrast-enhanced computed tomography (CT) scan of the neck revealed a 56 × 37 mm giant irregular mass in the left lobe of the thyroid gland, which involved adjacent structures and compressed the trachea and esophagus (Fig. 2A). The whole mass wrapped and compressed the esophagus, causing significant esophageal stenosis and resulting in dysphagia. Magnetic resonance imaging (MRI) revealed similar results and further confirmed the boundary between the tumor and the trachea, as well as esophageal compression and invasion (Fig. 2B). Esophagogastroduodenoscopy presented stenosis of the upper esophagus with no passage of the nasal gastroscope (Fig. 2C). The esophagram showed that the esophageal mucosa was less smooth locally, and the left esophageal wall was seemingly destroyed. Therefore, it could be concluded that significant compression and wall invasion of the upper esophagus existed, but there was no marked cervical lymphadenopathy.

**Figure 3:**
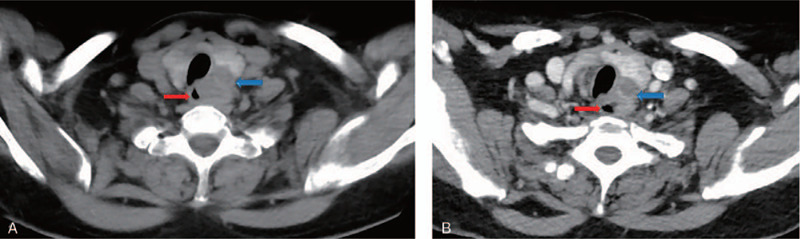
CT imaging demonstrating the improvement of esophageal compression and tumor reduction. (A) Scan after 1 month of apatinib. (B) Scan after 6 weeks of apatinib. CT = computed tomography.

The multi-disciplinary team (MDT) suggested that it was difficult to resect the tumor completely and safely due to the severe involvement of the surrounding structures of the trachea and esophagus. It might become a palliative operation if surgical treatment is selected as the primary choice, and the risk of esophageal fistula could also be high. Considering the potential antineoplastic activity and rapid response of apatinib in several kinds of malignancies, our team proposed off-label preoperative targeted therapy for this specific case. Of course, economic factors cannot be ignored: the cost of apatinib is only one-half that of sorafenib, and apatinib is also more available than sorafenib in China. Treatment with 500 mg apatinib orally once per day was started on February 14, 2019. Fortunately, the only adverse drug reaction that occurred was mild hypertension. One month after therapy, the patient benefited from favorable clinical tolerability and safety, and an interval CT scan indicated tumor shrinkage to 36 × 25 mm in size and a significant reduction in the degree of pressure on the esophagus (Fig. 3A). At the same time, the serum Tg level decreased from 1364.00 to 861.20 ng/mL. These surprising findings seemed to be a good sign of disease remission.

After the continuous administration of apatinib for 6 weeks, the patient felt remission of dysphagia. We reappraised the illness by a contrast-CT scan on April 1, 2019, and the results showed the size of the tumor was 29 × 26 mm, which was a marked reduction (Fig. 3B). In this circumstance, we believed that surgery could be less traumatic, complications could be more manageable, and the esophagus could be preserved. Then, total thyroidectomy, left central lymph node dissection, and muscular layer resection of the esophagus were smoothly completed. It is worth mentioning that the tumor had seriously invaded the left RLN. In addition, no invasion or adhesion to the trachea was discovered. Postoperative pathology and immunohistochemistry revealed a classic PTC diagnosis with capsular invasion comprising CK19(+), MC(+), galectin-3(+), TPO(–), TG(weak+), TTF-1(+), Ki-67 (5–10%), and PAX8(+) at the left lobe of the thyroid, and nodular goiter in the right lobe. In total, 1/2 of the lymph nodes in the central compartment were metastatic without extranodal extension. The final TNM staging was pT4aN1aM0, stage III. It is necessary to emphasize that concurrent BRAF V600E and TERT C228T mutations were found in tumoral tissue by genetic analysis of next-generation sequencing, which indicated the aggressive behavior of this PTC. We paid close attention to the recovery of the esophagus and found no esophageal fistula.

**Figure 4:**
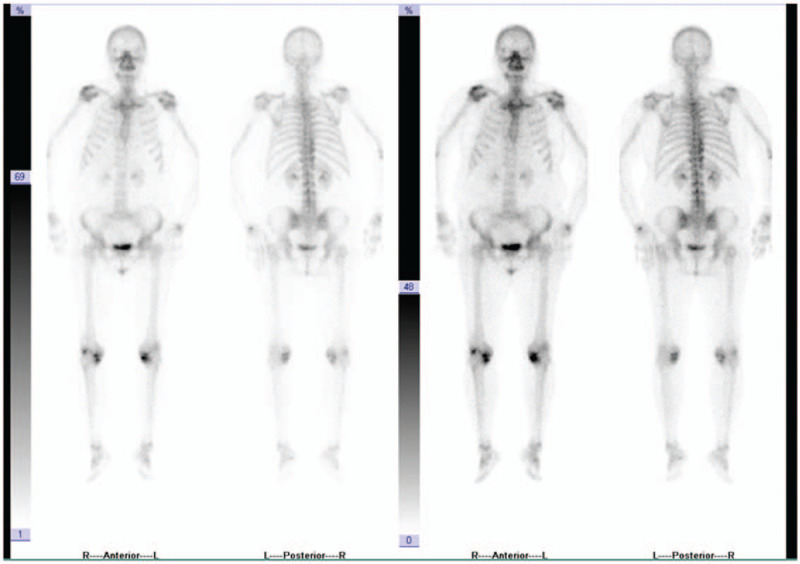
Whole-body scan finding no tumor bone metastasis 6 months after the operation.

**Figure 5:**
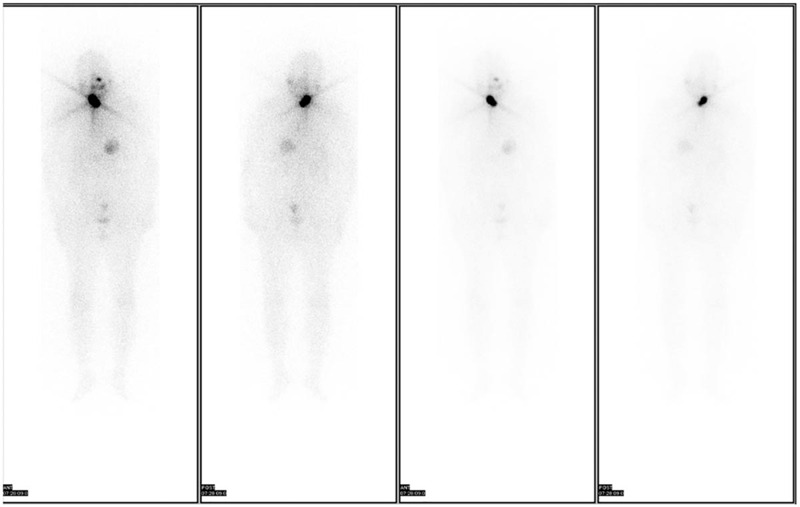
Whole-body RAI scan revealing focal ^131^I uptake in the cervical thyroid bed. RAI = radioactive iodine.

The level of Tg decreased to 4.80 ng/mL with anti-Tg antibodies (-) during the first month of follow-up. The level of TSH was adjusted to <0.1 mU/L by levothyroxine dosing to reduce the risk of disease recurrence. Six months later, neck ultrasonography found no residual TC or normal thyroid tissue, chest CT scan and whole-body bone scan (Fig. 4) revealed no pulmonary or bone metastasis, while whole-body RAI scan (Fig. 5) indicates focal ^131^I uptake in the cervical thyroid bed. Then, the patient underwent RAI remnant ablation with an administered activity of 100 mCi. One year after the surgery, the Tg level was 0.81 ng/mL, and the TSH level was 0.02 mU/L. In the future, long-term follow-up to evaluate postoperative disease status is still necessary.

## 3 Discussion

Locally advanced TC generally refers to gross extrathyroidal extension (gETE) of the primary tumor that passes the thyroid capsule and infiltrates the surrounding tissues. According to the Cancer Staging Manual of 8th edition American joint Committee on cancer (AJCC), gETE into subcutaneous soft tissues or the larynx, trachea, esophagus, or recurrent laryngeal nerve from a tumor of any size is defined as the T4a category, and the presence of gETE invading the prevertebral fascia, encasing the carotid artery or mediastinal vessels is T4b.^[10]^ Extensive gETE portends an increased incidence of local recurrence and regional and distant metastasis.^[3]^ Though management is controversial, it is certain that the most significant approach is to remove the neoplasm en bloc to achieve local control and extend disease-free survival. However, the balance between radical surgery and the functional preservation of the anatomical structures involved must be taken into account.^[11]^ There is no doubt, that surgical therapy for locally advanced DTC is challenging, and the resection scope remains controversial due to the lack of high-level evidence.

Research shows that free margins (R0) have better local control^[12]^ than close or positive microscopic margins (R1), while incomplete resection has been associated with increased mortality.^[5,13]^ In addition, lower local control was observed in recurrent tumors versus initial tumors, and the contribution of postoperative adjuvant radiation therapy was limited.^[12]^ In conclusion, attaining an R0/R1 resection by comprehensive operative clearance is critical and is associated with superior outcomes in T4 disease. In this case, demolitive surgery with reconstruction of the esophagus not only considerably aggravated the notable surgical trauma and postoperative complications but also significantly decreased the patient's quality of life. Above all, the patient's acceptance to undergo such procedures seemed vacillating.

In this particular case, which methods can we try in order to reduce the tumor volume to improve the resection margins and narrow the scope of surgery on the basis of R0/R1 resection? Neoadjuvant therapy for thyroid cancer still has not had an established role and is not mentioned in the 2015 American Thyroid Association Management Guidelines.^[3]^ Historically, only a few similar cases were found on review of the literature. Besic et al^[14,15]^ reported that preoperative ChT decreased the tumor size by >50% in 45% of patients (13/29) with FTC or HCTC and in 44% (7/16) of patients with locally advanced PTC. A case published in 1998 reported that preoperative ^131^I treatment is beneficial for inoperable TC.^[16]^ In addition, one locally unresectable patient underwent a total thyroidectomy after neoadjuvant EBRT.^[17]^ Nonetheless, a large number of studies have focused on targeted therapies, especially TKIs. For instance, surgical clearance was achieved in patients with locally advanced DTC after 14 months of treatment with sorafenib and lenvatinib,^[18]^ after 13 months of sorafenib monotherapy,^[19]^ or after 22 weeks of lenvatinib monotherapy.^[20]^ Lenvatinib was used to achieve better local control of PDTC during the waiting period for surgery because of patient comorbidities.^[21]^ A similar situation was observed in medullary thyroid cancer (MTC) with sunitinib^[22]^ or even in anaplastic thyroid cancer (ATC) with dabrafenib plus trametinib.^[23]^ Currently, vandetanib and cabozantinib are approved by FDA for patients with progressive, metastatic, or unresectable MTC, while sorafenib and lenvatinib are approved for patients with RAI-refractory DTC. All these findings reveal that neoadjuvant therapy could potentially reduce the extent of invasion and risks of residual disease after surgical resection. In certain cases, it might be a valuable option when treatment is limited.^[24]^

VEGF is a crucial and positive regulator of the physiological angiogenesis processes, which is essential for tumor growth, invasion, and metastasis. VEGFR-2, mainly expressed on endothelial cells, is the key signaling receptor of the pathway mediating the mitogenic, angiogenic, and permeability-enhancing effects of VEGF. Thus, therapies targeting VEGF or VEGFR mainly focus on anti-angiogenesis. Apatinib, also known as Aitan (brand name in China) and developed independently by Shanghai Hengrui Pharmaceutical Co., Ltd. (Shanghai, China),^[25]^ is a typical representative anti-angiogenesis agent with antineoplastic functions. It could induce apoptosis and suppress tumor proliferation across a variety of advanced solid malignancies with proven efficacy and safety for solid tumors,^[26–31]^ even TC.^[32,33]^ Of note, previous studies from our hospital also found that apatinib had better efficacy, a more manageable safety profile and faster therapeutic responses.^[9,34]^

In this case, the treatment of apatinib (orally 500 mg qd)^[33]^ showed a significant effect and fewer complications in a very short time, consistent with the findings of previous research.^[32]^ Just after treatment for 6 weeks and drug withdrawal for 10 days, R0 resection was accomplished. Tg detection and CT scans were performed to reassess the disease. Fortunately, there were no postoperative complications, such as wound dehiscence or fistula formation. At present, the use of TKIs is limited to thyroid cancer cases that still progress after surgery, radioiodine, or local ablation therapies. There are no data to certify the use of antiangiogenic TKIs as neoadjuvant therapy in DTC. Thus, there are still many issues, including drug categories, dosage, time of adding medicine, withdrawal time before surgery, methods of disease reassessment, and combination with conventional therapies, that are worthy of further study.

To our knowledge, this is the first report that preoperative monotherapy with the VEGFR-2 TKI apatinib was effective, rapid, safe and economical for unresectable locally advanced DTC. The emergence of this neoadjuvant concept has changed the landscape of the management of locally advanced TC. We believe that this study provides a new idea for the treatment of inoperable DTC cases to achieve extensive resection of extrathyroidal tissues and simultaneously improve quality of life. However, more cohort studies, randomized controlled trials, and even related mechanistic research are needed.

## Author contributions

**Resources:** Xianzhao Deng, Jie Kang, Bo Wu, Bomin Guo.

**Writing – original draft:** Yingchao Zhang.

**Writing – review & editing:** Zheng Ding, Youben Fan.
